# Applying Intraoperative Portal Venography in Liver Transplantation Vascular Surgery

**DOI:** 10.3390/diagnostics15182321

**Published:** 2025-09-12

**Authors:** Szu-Kai Wang, Yu-Fan Cheng, Wei-Xiong Lim, Chao-Long Chen, Leung-Chit Tsang, Chun-Yen Yu, Hsien-Wen Hsu, Po-Hsun Huang, Chun-Hua Chiu, Hsin-You Ou

**Affiliations:** 1 Department of Diagnostic Radiology, Kaohsiung Chang Gung Memorial Hospital, 123 Ta-Pei Road, Niao-Sung, Kaohsiung 83305, Taiwan; david455209@gmail.com (S.-K.W.); prof.chengyufan@gmail.com (Y.-F.C.); rahxaphon01@gmail.com (W.-X.L.); leolctsang@gmail.com (L.-C.T.); y7192215@ms17.hinet.net (C.-Y.Y.); lordblue607@yahoo.com.tw (H.-W.H.); qwe79103@gmail.com (P.-H.H.); photododophotododo@hotmail.com (C.-H.C.); 2 Department of Surgery, Kaohsiung Chang Gung Memorial Hospital, 123 Ta-Pei Road, Niao-Sung, Kaohsiung 83305, Taiwan; clchen@adm.cgmh.org.tw

**Keywords:** Intraoperative Portal Venography (IOPV), liver transplantation, portal vein thrombosis

## Abstract

**Background/Aim:** Adequate portal inflow is essential for liver graft regeneration following transplantation. Intraoperative portal venography (IOPV) provides real-time assessment of portal vein patency, stenosis, thrombus formation, and portosystemic collaterals. In addition to imaging, portal vein pressure gradient (portal vein pressure minus inferior vena cava pressure) was also measured. This study assessed the impact of IOPV on surgical decision-making and post-transplant outcomes to establish criteria for patient selection. **Methods:** From November 2016 to November 2024, 34 liver transplant patients with portal inflow insufficiency (flow velocity < 10 cm/s), large shunts (>1 cm), or portal vein thrombosis underwent IOPV. Of the patients, one received deceased donor liver transplantation (DDLT), and the others received living donor liver transplantation (LDLT). Preoperative computed tomography (CT) and ultrasound (US) assessed portal vein patency, thrombus, and shunts. Postoperative US and CT monitored portal flow and graft regeneration. **Results:** IOPV influenced surgical planning in all cases, leading to shunt ligation or stenting, and improved portal vein flow velocity from 6.3 (IQR, 0–9.0) to 30.8 (IQR, 22.2–36.7) cm/s (*p* < 0.001). Adequate inflow was achieved in 32 patients, 2 had persistent low flow or occluded flow owing to severe adhesion after transplant and failure to close large collateral veins. Graft regeneration ranged from 104% to 255% within a year. **Conclusions:** IOPV is a valuable tool in liver transplantation vascular surgery, optimizing surgical strategies and portal inflow. Early integration into routine practice may improve graft outcomes. Further prospective, longitudinal research is needed to refine patient selection and assess long-term benefits.

## 1. Introduction

Adequate portal inflow plays a crucial role in the regeneration of liver grafts following liver transplantation [1,2,3]. Therefore, having a reliable tool to monitor the portal vein is of utmost importance. Intraoperative ultrasonography is generally considered the first-line modality for assessing vascular patency and hemodynamics because of its non-invasive nature and real-time capability. However, in selected situations where ultrasonography is inconclusive or insufficient, intraoperative portal venography (IOPV) may serve as a complementary tool. IOPV provides direct and effective visualization of portal venous anatomy, not only enabling assessment of patency and detection of stenosis or thrombus formation but also facilitating identification of portosystemic collaterals [4,5]. Furthermore, it aids in surgical planning and facilitates subsequent interventional treatments, such as coronary vein or splenorenal shunt ligation and portal vein stent placement, to optimize graft perfusion and improve post-transplant outcomes [6,7,8,9]. Typical indications for IOPV include portal inflow insufficiency, large portosystemic shunts, or portal vein thrombosis. Nonetheless, its invasive nature limits its application to selected cases where additional hemodynamic assessment is essential.

Despite its potential, studies investigating IOPV remain relatively scarce or small in adult cases [4]. Thus, this study aimed to analyze and consolidate data from our institution over the past several years regarding liver transplant recipients who underwent IOPV. The specific objectives of this study were to assess the impact of IOPV on surgical decision-making and clinical outcomes, as well as to establish preliminary criteria for identifying patients who would benefit most from its use based on preoperative screening. Additionally, the study aimed to ascertain the optimal timing for performing portal venography for these patients.

## 2. Patients and Methods

### 2.1. Study Design and Sample

**Figure 1:**
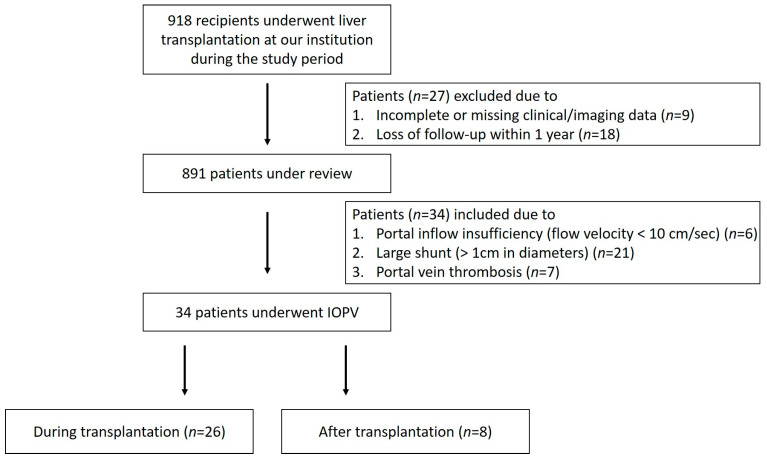
Flow chart of patient enrollment.

Between November 2016 and November 2024, a total of 918 recipients underwent liver transplantation at our institution. Among them, 34 patients (3.7%) underwent IOPV, either during (26 patients) or after (8 patients) transplantation (Figure 1). Of these, one received deceased donor liver transplantation (DDLT) with a split right lobe graft, and the others received living donor liver transplantation (LDLT). All patients underwent preoperative imaging, including Doppler ultrasound (US) approximately two weeks prior to transplantation and contrast-enhanced computed tomography (CT) approximately one month prior to surgery. These modalities were utilized to assess portal vein patency, evaluate for thrombus formation, measure flow velocity, and identify the presence of large portosystemic shunts or dilated coronary veins. Postoperatively, Doppler US was performed routinely to assess portal vein patency and flow dynamics. Furthermore, within the first year after transplantation, CT imaging was conducted to monitor graft regeneration by measuring its volume changes. Liver volume was determined using CT volumetric measurements. Graft weight was calculated as the product of 1.00 g/mL and CT-based graft volume [10]. Follow-up graft weight divided by the original graft weight was used to determine the liver graft regeneration rate.

The indication for performing IOPV was generally established preoperatively or intraoperatively in patients with evidence of portal inflow insufficiency (flow velocity < 10 cm/s), the presence of large portosystemic shunts (>1 cm in diameter, such as splenorenal shunt or coronary vein), or portal vein thrombosis. Post-transplant patients who underwent IOPV were also those found to have portal inflow insufficiency or large portosystemic shunts during follow-up assessments. These patients received IOPV and further interventional management, including portal vein stenting or coronary vein ligation to optimize portal hemodynamics and improve graft function. The impact of IOPV on surgical decision-making was analyzed, and improvement of portal inflow and subsequent graft regeneration were assessed after interventional management. Additionally, in order to ascertain the optimal time to perform IOPV and the interventional management that should be administered, the outcomes of patients who received IOPV during liver transplantation were compared with those who received postoperative IOPV.

### 2.2. IOPV Procedure

#### 2.2.1. Indication

Portal inflow insufficiency (flow velocity < 10 cm/s), large shunt (>1 cm in diameters) (splenorenal shunt, coronary vein), or portal vein thrombosis.

#### 2.2.2. Approach and Intervention Management

Step 1: The procedure began with vascular access, in which a mesenteric vein was identified, and a 6Fr angiographic sheath (Terumo, Tokyo, Japan) was inserted under direct visualization. Portal venography was subsequently performed by injecting approximately 20 mL of contrast medium through the angiographic sheath, enabling real-time visualization of the portal vein’s anatomy, patency, portal flow direction and the presence of significant portosystemic collaterals, such as coronary veins or splenorenal shunts. When small coronary veins were difficult to locate surgically, a guidewire was introduced into the vein via the catheter to serve as a marker for precise localization and ligation.

Step 2: The portal vein pressure gradient (portal vein pressure minus IVC pressure) was measured to assess portal inflow adequacy and the hemodynamic impact of collateral circulation [11]. If large portosystemic shunts were identified, they were selectively clamped to redirect blood flow into the liver graft. After clamping, the pressure gradient was measured. If the pressure gradient was less than 15 mmHg, the surgeon proceeded with shunt ligation; conversely, if it was more than 15 mmHg, the collateral vein was preserved to avoid small-for-size syndrome [12,13]. As an alternative to surgical ligation, embolization of collateral veins—such as coronary vein embolization using coils (Tornado, Cook Medical, Bloomington, IN, USA; Concerto, Medtronic, Minneapolis, MN, USA; Interlock, Boston Scientific, Marlborough, MA, USA) and intravascular glue (N-butyl cyanoacrylate, NBCA, Histoacryl, H.B. Fuller, Hudson, NC, USA)—was also considered.

Step 3: In patients with portal vein stenosis or anastomotic site stricture, venoplasty (Mustang, Boston Scientific, Marlborough, MA, USA) and stent insertion (E-Luminexx, Bard Peripheral Vascular, Tempe, AZ, USA) were performed as substantial management [14,15,16]. Following the intervention, a repeat IOPV was carried out to confirm restoration of adequate hepatic inflow and to ensure elimination of residual collateral circulation.

### 2.3. Statistical Analysis

Normality was assessed with the Shapiro–Wilk test, which showed non-normal distributions. Continuous variables were presented as median (IQR) and categorical variables as *n* (%). Paired continuous data were compared with the Wilcoxon signed-rank test, and subgroup differences were assessed using the Mann–Whitney U test. Categorical variables were analyzed with Fisher’s exact test. All tests were two-tailed, with *p* < 0.05 considered statistically significant. No missing data were present. Analyses were performed using SPSS version 20.0 (IBM).

## 3. Results

### 3.1. Patient Characteristics

Among the 34 patients who underwent IOPV, the median age was 55.5 (IQR, 50.8–60.3) years, with a male-to-female ratio of 10:7 (Table 1). The predominant etiologies in our cohort were cirrhosis related to hepatitis B virus (HBV), hepatitis C virus (HCV), and alcohol use, as well as hepatocellular carcinoma (HCC). The median model for end-stage liver disease score (MELD) was 15 (IQR, 11–18.5). Preoperative CT imaging identified large portosystemic shunts (>1 cm in diameter) in 21 patients, while preoperative US and CT detected portal vein occlusion in 13 patients.

### 3.2. Impact of IOPV on Surgical Decision-Making and Portal Flow

Notably, IOPV influenced surgical planning and decision-making in all cases, leading to interventions such as coronary vein or splenorenal shunt ligation or portal vein stent placement. These types of management ensure adequate portal inflow and optimize hepatic graft perfusion, thereby improving post-transplant hemodynamic stability and graft function. A statistically significant improvement of portal vein flow velocity was observed following IOPV and associated interventions, with an increase from 6.3 (IQR, 0–9.0) to 30.8 (IQR, 22.2–36.7) cm/s (*p* < 0.001). Of the 34 patients, 32 achieved sufficient portal perfusion post-intervention with mean portal vein flow velocity, while two patients with large coronary veins continued to experience persistently low or occluded portal vein inflow.

### 3.3. Graft Regeneration and Patient Survival

Within six months to one year of follow-up, liver graft regeneration ranged from 104% to 257% (median: 179%) of the initial graft weight. Additionally, graft size reached between 60% and 92% (median: 76%) of the estimated standard liver volume (SLV), which was calculated based on body surface area (BSA) [17,18]. The overall patient survival in this cohort was 100% at the latest follow-up in July 2025.

### 3.4. Subgroup Analysis: Intraoperative vs. Postoperative IOPV

Comparison between patients who underwent IOPV during transplantation (*n* = 26) and those after transplantation (*n* = 8) revealed no significant differences in baseline characteristics such as age, gender, MELD score, or pre-treatment portal vein flow velocity (Table 2). Additionally, patients who underwent IOPV, along with surgical and interventional management during liver transplantation, achieved a 100% portal vein patency rate postoperatively, and the longest follow up patient lasted for 9 years. Moreover, post-procedural assessments revealed a significant difference in portal vein flow velocity between these two groups. Patients who underwent IOPV during liver transplantation had an average flow velocity of 32.6 (IQR, 27.6–37.9) cm/s, whereas those who received IOPV after transplantation had a lower velocity of 24 (IQR, 8.4–29) (*p* = 0.010). Despite these differences in flow dynamics, no statistically significant differences were observed in graft regeneration rates between these two groups.

### 3.5. Representative Case

**Figure 2:**
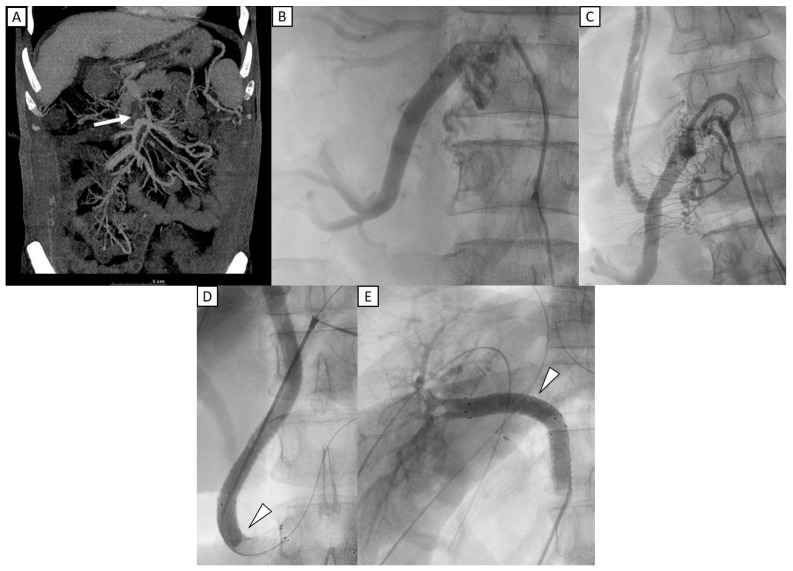
A 48-year-old man who underwent liver transplantation. (**A**) Preoperative CT imaging revealed total occlusion of portal vein with thrombosis of the superior mesenteric vein (SMV) (white arrow). (**B**) IOPV through the mesenteric vein confirmed complete occlusion of the distal SMV, with no flow to portal vein, and with a large ileocolic vein. (**C**) An interposition graft with Gelsoft (10 mm, 25 cm) (Terumo, Tokyo, Japan) was placed from the ileocolic vein to the grafted right portal vein. Multiple filling defects were identified within the interposition graft, favoring thrombi. (**D**,**E**) Two stents (12 mm × 60 mm) (E-Luminexx, Bard Peripheral Vascular, Tempe, AZ, USA) were deployed, with one extending from ileocolic vein to the interposition graft and another from the interposition graft to the grafted right portal vein (arrowhead). Post stenting portal venography confirmed patency of the interposition graft and portal vein.

Figure 2 shows a 48-year-old man who underwent liver transplantation. Preoperative CT imaging revealed thrombosis of the superior mesenteric vein (SMV), partial thrombosis of the main portal vein (MPV), dilated coronary veins, and an extrahepatic shunt originating from an engorged ileocolic vein. IOPV confirmed complete occlusion of the distal SMV and portal vein, with a large ileocolic vein. To reconstruct the portal vein, a long interposition graft with Gelsoft (10 mm, 25 cm) (Terumo, Tokyo, Japan) was placed from the ileocolic vein to the grafted right portal vein [19]. To optimize portal inflow, the distal end of the ileocolic vein was ligated. The follow-up IOPV demonstrated portal vein patency; however, multiple thrombi were identified within the interposition graft. Consequently, thrombectomy was performed, followed by venoplasty and stent placement. Two stents were deployed—one extending from the ileocolic vein to the interposition graft and the other from the interposition graft to the grafted right portal vein. Portal venography confirmed restored patency of both the interposition graft and the portal vein. Subsequent Doppler ultrasound evaluation demonstrated adequate portal perfusion, with a measured portal vein flow velocity of 32.1 cm/s.

## 4. Discussion

Among the 34 patients in this study, IOPV significantly influenced intraoperative decision-making, leading to interventions such as coronary vein and splenorenal shunt ligation or portal vein stent placement. These procedures are essential in optimizing portal inflow, ensuring adequate graft perfusion, and stabilizing post-transplant hemodynamics [6,7,8].

Portal vein flow velocity increased significantly following IOPV and the corresponding interventions, underscoring their effectiveness in restoring sufficient portal inflow, a critical factor for graft regeneration and function. Adequate inflow was achieved in 32 patients, while 2 continued to exhibit low or occluded flow despite intervention. Both of these patients had IOPV performed post-transplant and presented with severe adhesions, complicating the effectiveness of intervention. One patient presented with multiple large portosystemic shunts, including portal-renal and portal-retroperitoneal shunts. Despite shunt clamping, venography revealed that the portal vein was not directly visualized; however, a prominent collateral branch from the superior mesenteric vein to the choledochal vein was identified, demonstrating good intrahepatic inflow. Consequently, a portal-renal shunt ligation was performed to redirect blood flow. The second patient exhibited significant portal vein stenosis, managed with stent placement and coronary variceal occlusion using a vascular plug [20]. Although portal flow initially improved, follow-up US showed relatively low portal inflow, probably due to extensive portosystemic collateralization and refractory shunting. These cases underscore the challenges of severe shunting and the need for further refinement in additional therapeutic strategies to enhance outcomes in similar clinical scenarios. As illustrated in Figure 2, this case highlights the complexity of managing extensive venous thrombosis in liver transplantation and underscores the role of IOPV in guiding intraoperative vascular reconstruction and optimizing hemodynamic outcomes [21].

Liver graft regeneration ranged from 104% to 255% of the initial graft size within six months to one year post-transplantation, reinforcing that adequate portal inflow achieved through IOPV-guided management positively impacts graft growth. Importantly, overall patient survival in this cohort was 100% at the latest follow-up in July 2025, which appears more favorable compared with earlier IOPV studies, further underscoring the potential benefit of IOPV-guided intraoperative decision-making. These findings emphasize the critical role of sufficient portal inflow as a key determinant of long-term graft viability [22].

Additionally, preoperative CT and ultrasound were instrumental in detecting potential vascular abnormalities, including portal inflow insufficiency, large portosystemic shunts and portal vein thrombosis, all of which influenced the decision to perform IOPV. While preoperative imaging provided valuable insights, IOPV enabled real-time intraoperative visualization of vascular flow dynamics, offering crucial information that was not always apparent on preoperative assessment. Compared with the previous literature on IOPV, our study contributes additional clinical evidence. Earlier reports, such as Moon et al., (2007) [4] and Uchida et al., (2021) [5], primarily highlighted the feasibility of IOPV in detecting portosystemic collaterals or assisting in technically challenging portal vein reconstructions, often in small case series or pediatric populations. In contrast, our study included a relatively larger cohort of 34 patients, offering more robust clinical data. Moreover, our findings extend these observations by demonstrating significant improvements in portal flow and favorable survival outcomes. We further defined specific indications—such as portal inflow insufficiency, large portosystemic shunts, and portal vein thrombosis—and incorporated portal pressure gradient measurements to guide intraoperative management. These aspects underscore the added value of IOPV as both a diagnostic and decision-support tool in complex liver transplantation settings. Nevertheless, this study has several limitations, including its single-center retrospective design, relatively small sample size, and potential selection bias.

## 5. Conclusions

IOPV is a valuable tool in liver transplantation, optimizing surgical strategies and improving portal inflow. Early integration into routine practice may improve graft outcomes. Given the observed benefits of IOPV, future research is needed, focusing on refining preoperative screening criteria to better identify patients who would derive the most benefit from this technique. Furthermore, long-term follow-up studies are necessary to evaluate whether IOPV-guided interventions contribute to improved overall graft survival and patient outcomes. By integrating IOPV into routine intraoperative assessment protocols, liver transplantation strategies can be further optimized in patients with pre-transplant PV occlusion or large portal systemic shunts to enhance surgical precision, improve graft perfusion and promote long-term graft regeneration.

## Figures and Tables

**Table 1:** Demographic and clinical characteristics of recipients.

Case	Age/Gender	MELD	Etiology	Type of Transplantation/Graft Used	Additional Procedure	Portal Vein Flow Velocity (cm/s) (Before Intervention)	Portal Vein Flow Velocity (cm/s) (Intraoperative Ultrasound)	Portal Vein Flow Velocity (cm/s) (After Intervention)
1	48/M	15	Alcoholic cirrhosis, HCV	LDLT/right lobe	Ligation of distal portion of iliocolic vein	6.5	24.6	32.1
2	23/M	11	Cryptogenic liver cirrhosis	LDLT/left lobe	Ligation of coronary vein, splenorenal shunt and splenectomy	0	-	31.4
3	53/M	17	Alcoholic cirrhosis, HBV, HCC	LDLT/right lobe	Ligation of coronary vein	0	28.6	36.5
4	55/M	18	Alcoholic cirrhosis	LDLT/right lobe	Ligation of mesenteric vein	10.8	26.5	27.4
5	59/M	11	Alcoholic cirrhosis	LDLT/right lobe	Ligation of splenorenal shunt	7.8	35	48.1
6	58/M	14	HBV, HCC	LDLT/right lobe	Ligation of coronary vein	0	20	20
7	57/F	11	HCV	LDLT/right lobe	Ligation of coronary vein	9.4	21.9	45.7
8	58/F	14	Alcoholic cirrhosis	LDLT/right lobe	Ligation of SMV-IVC shunt	10.1	35.2	57.4
9	19/M	20	Biliary atresia	LDLT/right lobe	Ligation of SMV-IVC shunt and coronary vein	8	18.4	30
10	19/M	24	Biliary atresia	LDLT/left lobe	Embolization of coronary vein	0	-	34.6
11	51/M	27	HBV	LDLT/right lobe	Ligation of coronary vein	21.4 ^b^	24.2	30.6
12	60/F	11	HCV	LDLT/right lobe	Ligation of splenorenal shunt	0	18.4	47.8
13	65/F	15	HCV, HCC	DDLT/split right lobe	Ligation of SMV-IVC shunt and coronary vein	5	9	30
14	68/F	10	HCV, HCC	LDLT/left lobe	Ligation splenorenal shunt and splenoectomy	6.7	0	7.5
15	62/F	21	Autoimmune hepatitis, HCC	LDLT/right lobe	Ligation engorged IMV	11.6	-	37.3
16	53/F	15	Primary biliary cirrhosis	LDLT/left lobe	Ligation of coronary vein	12.7	10.2	38.2
17	64/M	13	HCV	LDLT/right lobe	Ligation of coronary vein and mesorenal shunt	5.2	24	33
18	61/F	17	HCV, HCC	LDLT/right lobe	Ligation of coronary vein and splenorenal shunt	7.7	15.9	30.9
19	62/M	10	Alcoholic cirrhosis	LDLT/left lobe	Ligation of coronary vein	7.1	34	27.6
20	55/F	12	HCV	LDLT/left lobe	Ligation of coronary vein	0	15.4	21.3
21	56/M	11	Alcoholic cirrhosis	LDLT/right lobe	Ligation of splenorenal shunt	0	-	0
22	50/F	17	HCV, HCC	LDLT/right lobe	Ligation of splenorenal shunt	0	23.4	35.4
23	62/M	15	Alcoholic cirrhosis	LDLT/right lobe	Ligation of splenorenal shunt	8.6	5.5	26
24	51/M	8	Alcoholic cirrhosis	LDLT/right lobe	Ligation of coronary vein and splenic artery	13.1	37.8	39.5
25	46/M	26	HBV	LDLT/right lobe	Ligation of coronary vein and short gastric vein	8.8	35.5	63.4
26	55/M	10	HCV, HCC	LDLT/right lobe	Ligation of coronary vein	0	0	11
27	60/M	18	Alcoholic cirrhosis	LDLT/left lobe	Ligation of coronary vein and splenic artery	8.6	26	35.4
28	54/F	25	HCV	LDLT/right lobe	Ligation of splenorenal shunt	0	13	26
29	54/M	14	HCV, HCC	LDLT/right lobe	Ligation of coronary vein and splenorenal shunt	0	17.3	21
30	64/M	16	Alcoholic cirrhosis, HCC	LDLT/left lobe	Ligation of coronary vein and splenorenal shunt	10.5 ^b^	16.3	22.2
31	49/M	14	HBV	LDLT/right lobe	RAPV thrombosis for thrombolysis	6	0	30
32	1/F	7 ^a^	Biliary atresia	LDLT/left lobe	SMV thrombosis for thrombolysis	0	-	22
33	57/F	17	HCV, HCC	LDLT/left lobe	Ligation of coronary vein	5.4	31.6	33
34	60/F	10	HCV	LDLT/left lobe	Ligation of coronary vein and portal vein stenting	0	7.6	21.2

^a^ Assessed by Pediatric End-stage Liver Disease (PELD) score. ^b^ Hepatofugal flow. Abbreviations: MELD, Model for End-stage Liver Disease; HBV, Hepatitis B virus; HCV, Hepatitis C virus; HCC, Hepatocellular carcinoma; LDLT, living donor liver transplantation; DDLT, deceased donor liver transplant; SMV, superior mesenteric vein; IVC, inferior vena cava; IMV, inferior mesenteric vein; RAPV, right anterior portal vein.

**Table 2:** Subgroup analysis with patients who underwent IOPV during and after liver transplantation (LT).

	During LT (*n* = 26)	After LT (*n* = 8)	*p*
Age (years)	56 (IQR, 49.8–60)	55.5 (IQR, 53.3–64.3)	0.537
Gender			
Male	17 (65.4%)	3 (37.5%)	0.228
Female	9 (34.6%)	5 (62.5%)	
MELD	14.5 (IQR, 11–18)	15 (IQR, 10–15)	0.450
Pre-treatment portal vein flow velocity (cm/s)	6.8 (IQR, 0–9.6)	2.5 (IQR, 0–8.1)	0.368
Post-treatment portal vein flow velocity (cm/s)	32.6 (IQR, 27.6–37.9)	24 (IQR, 8.4–29)	0.010 *

* *p* < 0.05.

## Data Availability

The raw data supporting the conclusions of this article will be made available by the authors on request.

## References

[1] Wu T.J., Dahiya D., Lee C.-S., Lee C.-F., Chou H.-S., Chan K.-M., Lee W.-C. (2011). Impact of portal venous hemodynamics on indices of liver function and graft regeneration after right lobe living donor liver transplantation. Liver Transplant..

[2] Marambio A., Tuñon J.M.C., Gómez L.M.M., Martínez J.M.A., Bellido C.B., Artacho G.S., Franco C.C., Pulido L.B., Ruiz F.J.P., Bravo M.A.G. (2018). Intraoperative Portal Vein Flow > 123 mL/min Per 100 g Predicts a Better Survival of Patients After Liver Transplantation. Transplant. Proc..

[3] Rasmussen A., Hjortrup A., Kirkegaard P. (1997). Intraoperative measurement of graft blood flow–A necessity in liver transplantation. Transpl. Int..

[4] Moon D.B., Lee S.-G., Ahn C., Hwang S., Kim K.-H., Ha T., Song G., Ryu J., Sung K., Ko G. (2007). Application of intraoperative cine-portogram to detect spontaneous portosystemic collaterals missed by intraoperative doppler exam in adult living donor liver transplantation. Liver Transplant..

[5] Uchida H., Sakamoto S., Shimizu S., Takeda M., Yanagi Y., Fukuda A., Abdelwahed M.S., Miyazaki O., Nosaka S., Kasahara M. (2021). Efficacy of intraoperative cine-portogram for complicated portal vein reconstruction in pediatric living donor liver transplantation. Pediatr. Transplant..

[6] Cheng Y.F., Ou H.-Y., Tsang L.L.-C., Yu C.-Y., Huang T.-L., Chen T.-Y., Concejero A., Wang C.-C., Wang S.-H., Lin T.-S. (2010). Vascular stents in the management of portal venous complications in living donor liver transplantation. Am. J. Transplant..

[7] Czerwonko M.E., Pekolj J., Mattera J., Peralta O.A., García-Mónaco R.D., de Santibañes E., de Santibañes M. (2019). Intraoperative stent placement for the treatment of acute portal vein complications in pediatric living donor liver transplantation. Langenbecks Arch. Surg..

[8] Kim Y.J., Ko G.-Y., Yoon H.-K., Shin J.-H., Ko H.-K., Sung K.-B. (2007). Intraoperative stent placement in the portal vein during or after liver transplantation. Liver Transplant..

[9] Ikegami T., Shirabe K., Nakagawara H., Yoshizumi T., Toshima T., Soejima Y., Uchiyama H., Yamashita Y.-I., Harimoto N., Maehara Y. (2013). Obstructing spontaneous major shunt vessels is mandatory to keep adequate portal inflow in living-donor liver transplantation. Transplantation.

[10] Celik H., Odaman H., Altay C., Ünek T., Özbilgin M., Egeli T., Ağalar C., Astarcıoğlu İ.K., Barlık F. (2024). Manual and semi-automated computed tomography volumetry significantly overestimates the right liver lobe graft weight: A single-center study with adult living liver donors. Diagn. Interv. Radiol..

[11] Reddy M.S., Rela M. (2017). Portosystemic collaterals in living donor liver transplantation: What is all the fuss about?. Liver Transplant..

[12] Ogura Y., Hori T., El Moghazy W.M., Yoshizawa A., Oike F., Mori A., Kaido T., Takada Y., Uemoto S. (2010). Portal pressure <15 mm Hg is a key for successful adult living donor liver transplantation utilizing smaller grafts than before. Liver Transplant..

[13] Hori T., Ogura Y., Ogawa K., Kaido T., Segawa H., Okajima H., Kogure T., Uemoto S. (2012). How transplant surgeons can overcome the inevitable insufficiency of allograft size during adult living-donor liver transplantation: Strategy for donor safety with a smaller-size graft and excellent recipient results. Clin. Transplant..

[14] Denys A., Chevallier P., Doenz F., Qanadli S.D., Sommacale D., Gillet M., Schnyder P., Bessoud B. (2004). Interventional radiology in the management of complications after liver transplantation. Eur. Radiol..

[15] Yang J., Xu M.-Q., Yan L.-N., Lu W.-S., Li X., Shi Z.-R., Li B., Wen T.-F., Wang W.-T., Yang J.-Y. (2009). Management of venous stenosis in living donor liver transplant recipients. World J. Gastroenterol..

[16] Zajko A.B., Sheng R., Bron K., Reyes J., Nour B., Tzakis A. (1994). Percutaneous transluminal angioplasty of venous anastomotic stenoses complicating liver transplantation: Intermediate-term results. J. Vasc. Interv. Radiol..

[17] Vauthey J.N., Abdalla E.K., Doherty D.A., Gertsch P., Fenstermacher M.J., Loyer E.M., Lerut J., Materne R., Wang X., Encarnacion A. (2002). Body surface area and body weight predict total liver volume in Western adults. Liver Transplant..

[18] Mosteller R.D. (1987). Simplified calculation of body-surface area. N. Engl. J. Med..

[19] Wu T.H., Lin Y.-S., Lee C.-F., Wu T.-J., Yu M.-C., Chan K.-M., Lee W.-C. (2011). Clinical analysis and strategy for liver transplantation in patients with pre-existing portal vein thrombosis. Chang Gung Med. J..

[20] Kim J.H., Ko G.-Y., Sung K.-B., Yoon H.-K., Kim K.R., Moon D.-B., Lee S.-G. (2009). Transvenous variceal embolization during or after living-donor liver transplantation to improve portal venous flow. J. Vasc. Interv. Radiol..

[21] Moon D.B., Lee S.-G., Ahn C.-S., Hwang S., Kim K.-H., Ha T.-Y., Song G.-W., Jung D.-H., Park G.-C., Namkoong J.-M. (2014). Section 6. Management of extensive nontumorous portal vein thrombosis in adult living donor liver transplantation. Transplantation.

[22] Matsuura T., Yoshimaru K., Yanagi Y., Esumi G., Hayashida M., Taguchi T. (2016). Insufficient Portal Vein Inflow in Children without Major Shunt Vessels During Living Donor Liver Transplantation. Ann. Transplant..

